# Lessons learned from conducting a serological survey for Japanese encephalitis after detecting the first cases in New South Wales, Australia, 2022

**DOI:** 10.5365/wpsar.2024.15.1085

**Published:** 2024-11-12

**Authors:** Zoe Baldwin, Sarah Davies, Kirsty Hope, Jennifer Case

**Affiliations:** a Master of Philosophy in Applied Epidemiology Programme, National Centre for Epidemiology and Population Health, The Australian National University, Canberra, Australia.; b Department of Health Protection, Hunter New England Local Health District, Newcastle, New South Wales, Australia.; c NSW Public Health Training Program, NSW Ministry of Health, St Leonards, New South Wales, Australia.; d NSW Ministry of Health, St Leonards, New South Wales, Australia.

## Abstract

**Problem:**

The first known locally acquired cases of Japanese encephalitis virus (JEV) infection in New South Wales (NSW), Australia, were identified in March 2022. NSW Health (the state entity for health care in NSW), with its partner agencies, conducted a serological survey to identify the prevalence of JEV antibody responses in high-risk communities in NSW.

**Context:**

JEV infection is rare in Australia; therefore, vaccination is not recommended for the majority of Australians. Less than 1% of JEV infections in humans result in clinical disease.

**Action:**

We conducted a cross-sectional serological survey of all age groups in five townships within NSW between June and July 2022. A summary report of the serosurvey methods and results was previously published by NSW Health. In this report, we describe the operations and lessons learned from rapidly gathering serological survey evidence to inform the public health management of JEV infection in NSW, within a country with well established health infrastructure.

**Lessons learned:**

Resource limitations had to be addressed pragmatically during this field epidemiology research. Community participation varied between towns. The knowledge of local public health staff was important for identifying appropriate locations for clinics and community engagement activities. The consistency of data collection needs to be emphasized when multiple teams are involved. Data quality assurance issues were limited during this survey, owing to ease of communication in the field with the coordinating research team. When possible, allowing additional time for community engagement and staff orientation would be beneficial before implementing a similar survey. Further consideration of reporting serology results during the study design stage might have prevented the need for manual processing upon study completion.

**Discussion:**

This serological survey highlights that a well trained and coordinated public health workforce can provide important, timely evidence when faced with an emerging public health issue.

## PROBLEM

Japanese encephalitis virus (JEV) was detected for the first time in New South Wales (NSW), Australia, in February 2022. (*1*) Only five human clinical cases of Japanese encephalitis had previously been reported in Australia, and this was the first known incursion of JEV south of the Cape York Peninsula. (*2*) By June 2022, NSW had recorded 13 confirmed cases of Japanese encephalitis, including two deaths. (*1*) Because there were few symptomatic cases, a cross-sectional serological survey was undertaken to better understand the outbreak in identified high-risk areas and to inform public prevention measures. We describe the operations and lessons learned from rapidly gathering serological survey evidence to inform public health management of JEV infection in NSW.

## CONTEXT

The introduction of JEV to NSW required a rapid and coordinated public health response to identify geographical risk areas and inform risk mitigation strategies. As JEV infection is rare in Australia, vaccination is not recommended for the majority of Australians. Prior to the outbreak, vaccination against Japanese encephalitis was recommended for travellers spending 1 month or more in endemic countries, people living and working in the outer islands of the Torres Strait, and laboratory workers who may be exposed to the virus. (*3*) Less than 1% of JEV infections in humans result in clinical disease. (*4*) As this was the first time that JEV was detected in NSW, there was little information that could be used to determine the extent of the outbreak or local risk factors for infection. Once Japanese encephalitis was declared a communicable disease incident of national significance, (*5*) NSW Health (the state entity for health care in NSW) rapidly implemented research and surveillance activities, including this serology study in high-risk areas.

## ACTION

### Implementing the serological survey

**Fig. 1:**
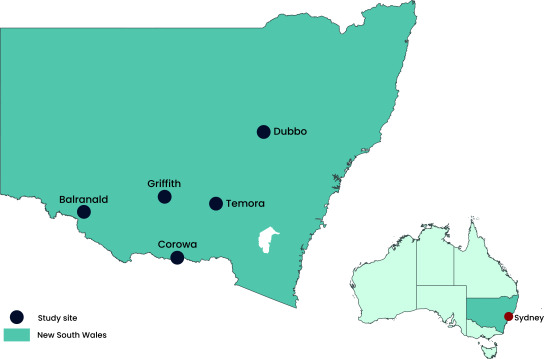
Map of study sites, New South Wales, Australia, June and July 2022

NSW Health, with its partner agencies, conducted a cross-sectional serological survey using convenience sampling in five high-risk towns in NSW (**Fig. 1**). The aim of the study was to estimate JEV antibody prevalence in communities that had limited evidence of transmission. Town selection was based on emerging data from animal and vector surveillance as well as consideration of logistics and resource limitations.

[**insert** Figure 1]

For logistical ease, the first three clinics for the survey were held in Balranald, Corowa and Temora at hospitals or associated health service locations. Clinics in Dubbo and Griffith were held in community centres. Additionally, mobile teams conducted outreach clinics in Dubbo (*n* = 8), Griffith (*n* = 13) and Temora (*n* = 2) at consenting business premises. The main outreach clinic in Griffith was conducted at a local shopping centre and required public liability insurance.

The first clinic was held in Corowa, as there had been local cases of Japanese encephalitis. Therefore, a significant level of interest in participating was anticipated due to high community awareness. The experience in Corowa informed subsequent clinics, allowing for refinement of clinic procedures and participant recruitment.

Serosurvey methods and results have been published elsewhere. (*6*) Briefly, the results indicated that 8.7% (80/917) of participants had evidence of JEV infection. Those aged ≥ 65 years showed the largest seropositivity proportion (30/192, 15.6%), and no participants aged < 20 years were seropositive. Participants from all five townships had evidence of infection.

### Participant recruitment and response

Staff from local public health units (PHUs) promoted participation in the study by engaging with local media (e.g. newspapers and radio stations), councils, general practitioners (GPs), local hospital staff, other government agencies (e.g. Local Land Services and police), businesses and community groups. Posters advertising the clinics were distributed to businesses. Targeted social media posts were also used.

**Fig. 2:**
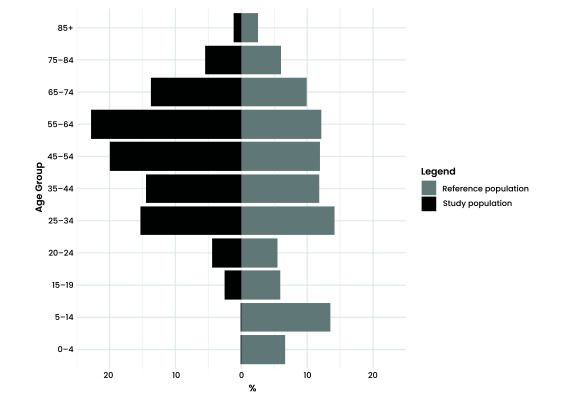
Population pyramid of study population and reference population, 2022

There were 1048 participants who completed a questionnaire and provided a blood sample, giving an overall response rate across the five towns of 1.2%, ranging from 0.7% in Dubbo to 4.4% in Balranald (**Table 1**). Overall, participants tended to be older (**Fig. 2**, with more females (*n* = 623) than males (*n* = 425) participating.

[**insert** Figure 2]

**Table 1:** Response rate for a community serology study of the prevalence of Japanese encephalitis antibody, by site, New South Wales, Australia, June and July 2022

Location	No. (%) of participants	Population size^a^ (% tested)
Balranald	64 (6.1)	1452 (4.4)
Corowa	163 (15.6)	7050 (2.3)
Dubbo	300 (28.6)	46 078 (0.7)
Griffith	362 (34.5)	28 126 (1.3)
Temora	159 (15.2)	6100 (2.6)
Total	1048 (100)	88 806 (1.2)

^a^ The population is derived from Australian Bureau of Statistics census mesh blocks within a 20-km radius of each town. (*7*)

### Staffing and logistics

The study was coordinated by Health Protection NSW (an entity of NSW Health), and the intention was that local PHU staff would conduct the clinics. However, the availability of local PHU staff to conduct clinics at all five study sites was limited; therefore, 48 staff from across the NSW Health network were assembled into multiple teams to travel to the study sites. Two primary roles were assigned in each team: operational support and blood collection.

All team members participated in a short virtual briefing, during which they could ask questions of the coordinating study team. A briefing document was provided about clinical protocols, background on the study design and information links to JEV factsheets and frequently asked questions. Communication between Health Protection NSW and field staff was maintained through Microsoft Teams, and daily debriefings were arranged, as necessary.

### Survey instruments and information technology

REDCap (*8*) software (Research Electronic Data Capture, Vanderbilt University, Nashville, TN, USA) was used to set up online consent forms and to collect demographic and exposure information from participants. Prior to their use, internal user testing was conducted to refine question phrasing and improve data quality. The survey was designed to be administered in the field using tablet devices, with support from operations staff as needed. Wi-Fi dongles were sent to field sites in case there were connectivity issues with fixed wireless internet connections.

In Griffith, where the largest number of workplace clinics took place, QR codes linking to the REDCap survey were printed, and many participants completed the surveys on personal smartphones.

### Laboratory testing and providing results

Laboratory samples were received via the usual transportation mechanisms. NSW has a strong laboratory infrastructure, and as a result, there were no challenges in ensuring blood samples were sent to and received by the reference laboratory. Results were provided to participants: those with positive results were notified by a phone call from a clinician; those with negative results were notified by SMS or e-mail. (*9*) Guidance was provided regarding the meaning of the results in relation to potential immunity and vaccination. (*9*) A copy of each participant’s results was sent to their GP, if requested, although this was done manually and not through the usual laboratory process, as details of the GP were not captured by the pathology request forms during blood collection.

## LESSONS LEARNED

### Implementing the serological survey

The availability of staff, particularly those with phlebotomy skills, was a significant constraint that impacted how the clinics were run. The clinics needed to be organized sequentially, in stand-alone locations that were separate from other pathology services, and over relatively short, fixed periods (i.e. 3–5 days during 1 week at each location). Clinics were held only on weekdays, as staff travelled to the study sites on weekends, which may have limited representativeness at some sites. School holiday periods were avoided to improve the chances of capturing residents and those employed in the townships.

After the first three clinics, staff identified limited public foot traffic at some locations because they were outside the town centres. Therefore, the clinics in Griffith and Dubbo were held in town community centres, albeit still with limited passing foot traffic. Promotional activities were important drivers of participation rather than foot traffic.

### Participant recruitment and response

The response rates in the smaller towns of Balranald, Corowa and Temora exceeded expectations and were higher than those in the larger towns of Dubbo and Griffith (**Table 1**). Many participants mentioned hearing about the clinic through traditional and social media, and they were also aware of, or connected to, local cases. Residents in the larger towns of Dubbo and Griffith appeared less aware of and engaged with JEV as a public health issue. It is possible that social connectedness and perceived proximity to risk in the smaller towns influenced the motivation to participate. (*10*)

The flexibility to adapt the approach to reach community members and encourage participation was important. Team feedback suggested that phone calls from and visits by clinic staff were helpful to explain the study at outreach locations and, along with ensuring more extensive stakeholder engagement 1–2 weeks before the clinic, may have further increased participation.

Towns where workplace clinics were organized had a greater proportion of younger, working-age participants, demonstrating that the locations used for the clinics influenced participation. Additional strategies could have been used to encourage participation among children, young people and Aboriginal and Torres Strait Islander peoples, who were underrepresented in the survey compared with the source population. Future approaches could consider developing demographic participation targets to increase representativeness and generalizability.

### Staffing and logistics

Having more time to prepare the clinics and to discuss site-specific considerations before opening might have been useful. However, time and travel constraints limited these opportunities at some sites. Nevertheless, the different teams reported that they worked well together to resolve issues as they arose, and they benefited from the support of local PHUs and the study coordination staff at Health Protection NSW.

The physical set-up in each clinic was different, and local support was variable. Detailed information about the space, equipment and access to consumables was not always available in advance, which made ordering clinic supplies challenging at times. It was also difficult to know in advance how many participants to plan for.

Organizing clinics primarily in one location versus at several workplace outreach locations presented a trade-off between increasing the numbers of participants and having enough staff, vehicles, information technology and blood collection equipment to support multiple locations. Ensuring that the teams at the clinics were using consistent practices became challenging across different locations, but it did result in greater numbers of participants. When the numbers of participants were high, staff had less time to clean data and support participants taking the survey. It was necessary to have a robust system to match a participant’s survey with their blood sample, particularly when there were many participants, and time often had to be allocated for staff to clean data every few hours.

### Survey instruments and information technology

Most participants successfully used the provided touchscreen tablets to complete the survey directly in the REDCap database, and clinic staff provided assistance as needed. A small number of surveys was completed on paper due to a lack of Wi-Fi access rather than to participant preference, with data subsequently entered by clinic staff. Refinements to the survey were made following the first clinic, and these might have been reduced by more extensive user testing before the clinics.

The use of QR codes to access the survey decreased the time spent at the clinic by participants, as they did not need to wait for tablet devices to become available. However, greater attention was required for those using the QR codes to manage data quality and ensure blood samples were correctly matched to survey records. Communication between team members in the field and at Health Protection NSW facilitated optimal data collection and quality assurance processes.

### Laboratory testing and providing results

It was an oversight not to ask for details about GPs on the pathology request forms during blood collection, and this meant that laboratory results systems could not be used to send results to GPs. Instead, workarounds and manual collation of the results were required to send results individually.

## Discussion

In NSW, an agile response by skilled and experienced researchers working in partnership with local PHUs and communities enabled the successful implementation of the first community-based cross-sectional serological survey for JEV infection, conducted with limited lead time. Maintaining a flexible approach enabled the team to overcome challenges as they arose. The lessons outlined in this report can be applied to other contexts and jurisdictions for similar operational research projects conducted during an outbreak.

There were substantial challenges to operationalizing the study ahead of the 2022–2023 summer season, as it was desirable to have results to inform public health response activities. The results were used to inform vaccination policy, public health prevention messaging and a communication campaign. In the context of an active outbreak response, our knowledge and understanding of JEV infection in south-eastern Australia was evolving. This posed challenges for designing the study.

A skilled public health workforce and collaborative health research approach resulted in timely evidence that could be used to inform the public health response to Japanese encephalitis in NSW. Leveraging stakeholder relationships with pathology services and with PHU directors and staff was key to rapidly conducting the study. Strong community engagement from local PHU staff was integral not only to respond to the local outbreak but also to build relationships to drive community participation in the serological survey. Central coordination worked well and field communications were maintained via digital technologies.
